# Using the Fossil Record to Evaluate Timetree Timescales

**DOI:** 10.3389/fgene.2019.01049

**Published:** 2019-11-12

**Authors:** Charles R. Marshall

**Affiliations:** ^1^Department of Integrative Biology, University of California, Berkeley, Berkeley, CA, United States; ^2^University of California Museum of Paleontology, University of California, Berkeley, Berkeley, CA, United States

**Keywords:** timetree, calibration, phylogeny, cladogram, fossil record, absolute time

## Abstract

The fossil and geologic records provide the primary data used to established absolute timescales for timetrees. For the paleontological evaluation of proposed timetree timescales, and for node-based methods for constructing timetrees, the fossil record is used to bracket divergence times. Minimum brackets (minimum ages) can be established robustly using well-dated fossils that can be reliably assigned to lineages based on positive morphological evidence. Maximum brackets are much harder to establish, largely because it is difficult to establish definitive evidence that the absence of a taxon in the fossil record is real and not just due to the incompleteness of the fossil and rock records. Five primary methods have been developed to estimate maximum age brackets, each of which is discussed. The fact that the fossilization potential of a group typically decreases the closer one approaches its time of origin increases the challenge of estimating maximum age brackets. Additional complications arise: 1) because fossil data actually bracket the time of origin of the first relevant fossilizable morphology (apomorphy), not the divergence time itself; 2) due to the phylogenetic uncertainty in the placement of fossils; 3) because of idiosyncratic temporal and geographic gaps in the rock and fossil records; and 4) if the preservation potential of a group changed significantly during its history. In contrast, uncertainties in the absolute ages of fossils are typically relatively unimportant, even though the vast majority of fossil cannot be dated directly. These issues and relevant quantitative methods are reviewed, and their relative magnitudes assessed, which typically correlate with the age of the group, its geographic range, and species richness.

## 1 Introduction

Developing rigorous methods for using paleontological and geological data to estimate divergence times between lineages has proven challenging. Yet, these methods are needed for both the construction and evaluation of timetrees (Donoghue and Yang, 2016), trees where the relative branch lengths are largely derived from DNA sequence data but have been converted into units of absolute time. Timetrees consist of a topology, branch lengths proportional to time, and an absolute timescale. Here, I am specifically interested in the paleontological evaluation of the timescales, the estimates of lineage divergence times—that is, I focus on how paleontologists estimate divergence times, not on how a given timetree might have been generated. Nonetheless, some of my discussion has bearing on the construction of timetrees, especially those derived from node-dating methods where the fossil record is used to provide priors on divergence times, including the difficult-to-establish maximum age constraints (Yang and Rannala, 2006; Ho and Phillips, 2009). Some of my discussion is also relevant to non-node-dating methods for constructing timetrees (see Donoghue and Yang, 2016 for a review), even though these do not require *a priori* maximum estimates of divergence times, for they still need to make assumptions about the rates of fossil recovery (Warnock et al., 2017). These methods include the Fossilized Birth Death (FBD) process (Heath et al., 2014; Stadler et al., 2018), total evidence methods that simultaneously estimate the phylogenetic position of the extant taxa and relevant fossils (Pyron, 2011; Ronquist et al., 2012), and integration of the FBD and total evidence methods (Zhang et al., 2016; Gavryushkina et al., 2017).

### 1.1 The Three Components of the Paleontological Estimation of Divergence Times

**Figure 1:**
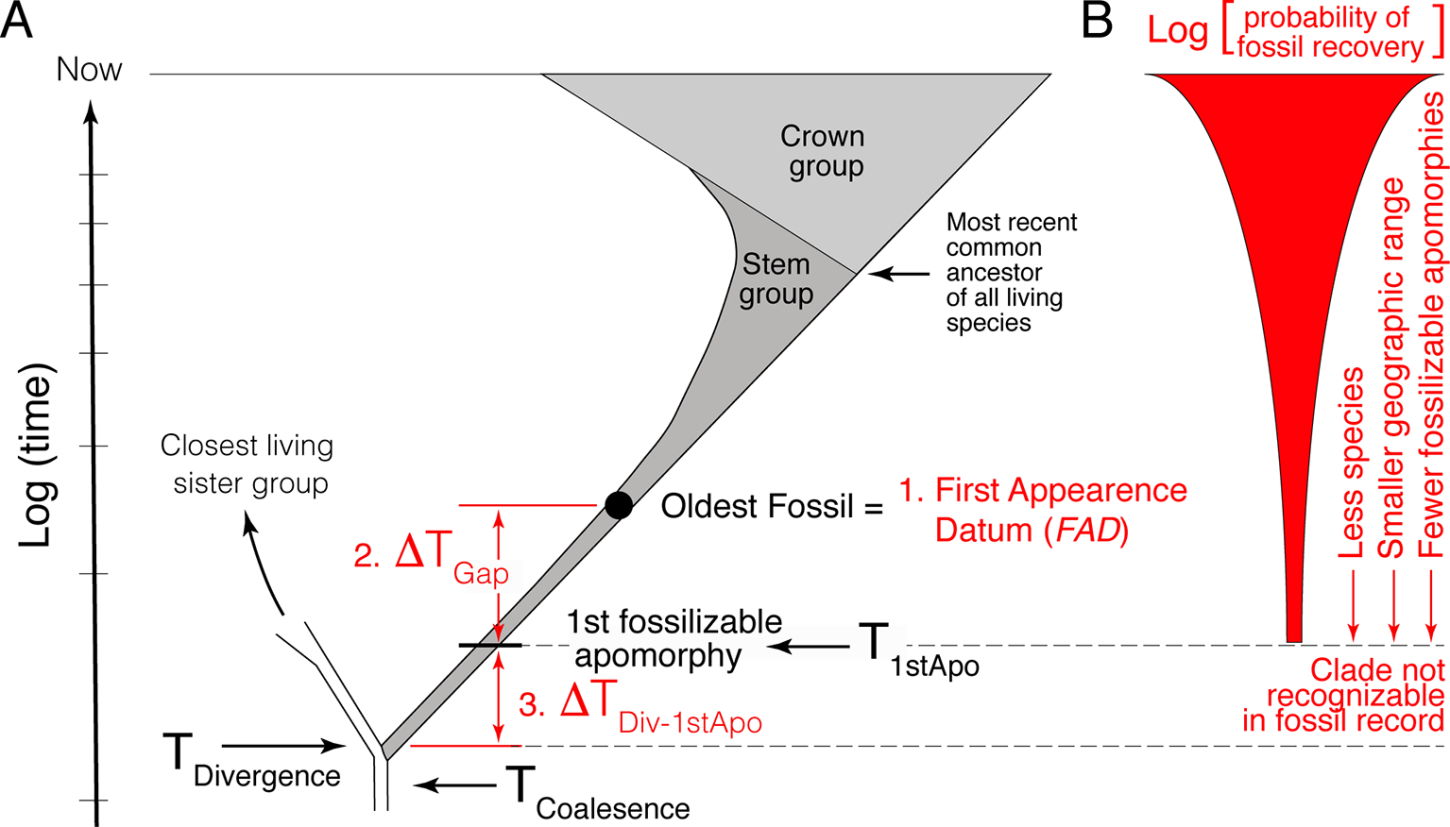
Schematic representation of the challenges encountered when using the fossil record to estimate divergence time between lineages. **(A)** The three primary challenges are: 1) the easiest, identifying and determining the age of the oldest fossil (*FAD*); 2) estimating the size of the temporal gap between the *FAD* and the time of origin of the first fossilizable apomorphy of the lineage (ΔT_Gap_); and 3) estimating the size of the gap between the true time of origin of the first fossilizable apomorphy and the actual divergence time (ΔT_Div-1stApo_). ΔT_Div-1stApo_ cannot be directly addressed with the fossil record because fossils that belong to this part of the clade’s history are not recognizable. **(B)** Estimating the size of ΔT_Gap_ is made difficult by the fact that the probability of recovering fossils for most lineages decreases as one approaches its time of origin, as well as the fact that the fossil and rock records are idiosyncratically incomplete temporally and spatially (not depicted).

The first component is the simplest, establishing the minimum estimate of the divergence time. This consists of identifying the oldest fossil of the focal lineage, its First Appearance Datum (*FAD*) (**Figure 1A**). As paleontologists are typically limited to working with morphological data, the minimum age constraint corresponds to the age of the oldest appearance in the fossil record of the first fossilizable apomorphy of the focal lineage.

Given the incompleteness of the fossil record, a literal reading of the fossil record is biased in that the age of the *FAD* will post-date the divergence time—we need to estimate the size of this temporal gap, that is, provide a maximum age constraint. However, because paleontologists must deal with morphological data, the statistical methods paleontologists have developed for estimating maximum age constraints actually pertain to the estimation of the true time of origin of the first fossilizable apomorphy (ΔT_Gap_ in **Figure 1A**) not the actual divergence time itself. Thus, estimating maximum age constraints consist of two steps. The first step, and second component of the paleontological estimation of divergence times, consists of estimating the size of the temporal gap between the *FAD* and the true time of origin of the first fossilizable apomorphy (ΔT_Gap_ in **Figure 1A**). The second step, and third component of the paleontological estimation of divergence times, consists of estimating the size of the gap between the true time of origin of this first apomorphy and the actual divergence time between the focal lineage and its extant sister clade (ΔT_Div-1stApo_ in **Figure 1A**). This last factor is often ignored, although it has long been recognized (e.g., see Marshall 1990b; Magallon, 2004; Steiper and Young, 2008; Marshall and Valentine, 2010). It is the hardest to quantify because there will typically be a lag between the time of genetic separation of two lineages, their divergence time, and the time of origin of the first fossilizable diagnosable morphological feature, the first autapomorphy, in the focal lineage. As discussed below, one or both of ΔT_Gap_ and ΔT_Div-1stApo_ can be large depending on the taxon.

### 1.2 Coalescence Times

Turning for a moment to the DNA component of timetrees, note that DNA data, when properly calibrated, provide a measure of the divergence time (T_Divergence_) plus the coalescence time for the loci being compared (e.g., see **Figure 1** in Edwards and Beerli, 2000) (**Figure 1A**):

(1) $${\text{T}}_{\text{DNA}}={\text{T}}_{\text{Divergence}}+{\text{T}}_{\text{Coalescence}}$$

Thus, even with accurate and precise temporal calibration with metronomically evolving DNA sequences, estimated divergence times will be too deep if one fails to take into account the standing polymorphism that was present at the time of the population divergence of the lineages of interest unless a correction has been made (e.g., Marshall and Swift, 1992). This issue is most important for shallower divergence times, typically less than a few million years, where the magnitude of the coalescence time can be a significant proportion of the divergence time (Edwards and Beerli, 2000). Note that this issue may be compounded by the fact that different loci may yield different topologies, which in turn may lead to incorrect branch lengths, which can impact inferred divergence times. But even if all loci yield the same topology, equation (1) still holds—DNA data bear directly on the coalescent time between the loci analyzed, not the divergence time *per se*.

### 1.3 The Challenge of Dealing With the Temporally Biased Fossil Record

Temporal information in the fossil record is biased, with correctly identified well-dated *FAD*s being younger than their respective divergence times. Quantifying how much older divergence times are than *FAD*s is challenging because there is no positive evidence that a taxon existed a given temporal distance beyond its know temporal (stratigraphic) range; it is hard to establish whether the absence of the taxon is real or just due to the incompleteness of the fossil record. Statistical approaches can be used, but the rigor of these approaches is made difficult by the fact that the probability of finding fossils of a clade generally decreases beyond its *FAD* (**Figure 1B**) given that: 1) at the time of inception of a clade there is only one lineage; 2) they likely lived in a limited geographic area; and; 3) typically, there are fewer and fewer diagnosable morphological features with, which to recognize fossils of the focal clade as one approaches its time of inception, a factor exacerbated by the fact that fossils are often fragmentary.

In the discussion that follows, I concentrate on quantitative methods where they exist. Note that the best practices depend in part on: 1) the richness of the fossil record within the focal group; 2) the richness of the fossil records of clades that lie outside the focal group with similar fossilization potentials; 3) the phylogenetic scope of the study; and, (4) the depth in geologic time over which the focal group evolved, which is typically correlated with the phylogenetic scope of the study.

#### 1.3.1 Heterogeneity in the Incompleteness of the Fossil Record

The stochastic nature of the fossil record means that the gap size between *FAD*s and true divergence times will be heterogeneous in size, which becomes relevant when generating timetrees with methods that use uncorrelated rates of molecular evolution (see section 1.4. below), and when contemplating the use of cross-validation approaches (see section 3.6 below). This heterogeneity has long been recognized (Jaanusson, 1976; Marshall, 1995), and its importance for the temporal calibration of molecular phylogenies was highlighted by Springer (1995). Springer (1995) showed using the Australian marsupial fossil record that a literal reading of the fossil record led to an estimate of the average rate of singly copy DNA evolution of 1% per million years, with a 17-fold difference from one lineage to the next (this was before DNA-branch lengths were used as part of timetree estimation). However, once Springer (1995) took into account the incompleteness of the fossil record using a confidence interval approach (see section 3.1 below), the data were shown to be consistent with a constant rate of DNA evolution at a much slower rate of 0.4% per million years.

### 1.4 Timetree Construction Is Especially Sensitive to Paleontological Data

It is well recognized that timetree timescales are very sensitive to the paleontological data used for calibration [e.g., see Barba-montoya et al. (2018) for a succinct summary]. Part of the reason is that when constructing timetrees there is typically no further explicit information on absolute time beyond the paleontological data used; thus, in Bayesian analysis for example, there is no direct data within the analysis to update the priors on the divergence times, and thus, those priors tend to constrain the range of dates in the resulting timetree. This dependence on the paleontological data means that timetree construction with uncorrelated rates of molecular evolution with priors that favor a literal reading of the fossil record (i.e., exponential priors; see Section 3.1.2 below) will tend to collapse the nodes onto the ages of the *FAD*s.

The sensitivity to the paleontological data itself stems from: 1) the difficulty in establishing rigorous maximum age constraints on divergence times [relevant to node-dating approaches (Yang and Rannala, 2006; Ho and Phillips, 2009)]; 2) the uncertainty in the phylogenetic placement of fossils either due to missing data or conflicting characters (e.g., see Sterli et al., 2013) (which effects almost all approaches); and, 3) uncertainties in the actual dating of fossils [which can have a large effect on total evidence approaches (O’Reilly et al., 2015)].

If this review has any simple take home message, it is that it is crucial that the utmost care be taken in specifying divergence time priors (Warnock et al., 2015).

## 2 Estimating Robust Minimum Divergence Times

The best practices for establishing minimum age estimates from fossil data, the oldest fossil securely assignable to the focal lineage, are well established (e.g., Benton and Donoghue, 2007; Donoghue and Benton, 2007; and especially Parham et al., 2012). Below, I outline the key points. I do not consider the use of paleobiogeographic constraints, except to note that they often lack precision both because the emergence of a land-bridge or the opening of a seaway is often a protracted event, and because most organisms have a dispersal capacity which means that divergence times can predate the formation of biogeographic barriers, often by an unknown magnitude. See Ho et al. (2015) and De Baets et al. (2016) for synoptic summaries of issues associated with using biogeographic constraints on divergence times, Loeza-Quintana and Adamowicz (2018) for an iterative approach for dealing with the complexity typical of biogeographic constraints, and Landis (2017) for a method for integrating information from multiple biogeographic constraints.

### 2.1 Minimum Times of Origin (*FAD*s) Must Be Apomorphy Based

This principle has now been well articulated (e.g., see Benton and Donoghue, 2007; Donoghue and Benton, 2007; Parham et al. 2012; Sauquet et al., 2012). Parham et al. (2012) also emphasize the importance of explicit listing of relevant museum numbers for the specimens that show the chosen apomorphies, as well as reconciling any discordance between molecular and morphological phylogenies that might impact which node the calibration fossil calibrates. It is important to take into account uncertainties in the phylogenetic position of calibration fossils, as these can greatly impact timetree calibration (Sterli et al., 2013). Careful selection of apomorphy-rich calibration fossils helps ameliorate the impact of this factor. I will not discuss here the interesting approaches designed to co-estimate the phylogeny of all the taxa, both the fossil and living (Pyron, 2011; Ronquist et al., 2012), where the phylogenetic placement of fossils is part of the process of generating the timetree, except to note that the richer and more accurate the morphological description of the fossils, the less ambiguity there will be in where those fossils join the phylogeny.

**Figure 2:**
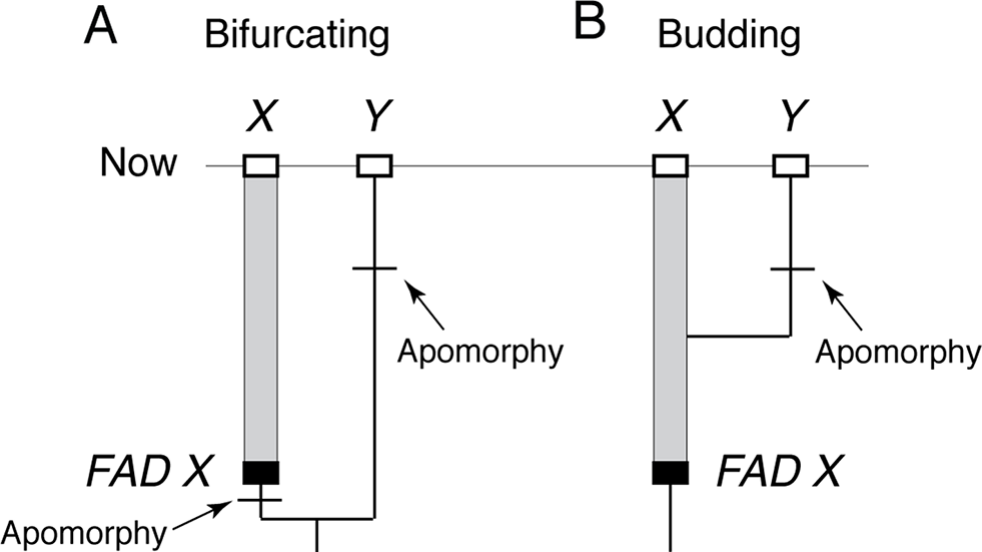
The importance of apomorphy-based *FAD*s when establishing minimum age constraints. **(A)** When lineage *X* has an apomorphy with respect to lineage *Y*, then oldest fossil of *X*, *FAD X*, can be used as minimum age constraint on the divergence of *X* and *Y*. **(B)** However, if *X* lacks apomorphies with respect to *Y*, then the age of *FAD X* does not constrain the divergence time of *X* and *Y* because *Y* could have budded off *X* at any time. Note: without a fossil record of *Y*, it is not possible to place a minimum age constraint on when its apomorphy evolved.

The reason *FAD*s must be apomorphy based is easily demonstrated. Imagine two closely related living taxa *X* and *Y*, where *Y* has morphological autapomorphies with respect to *X*, and where there is an oldest fossil that belongs unequivocally to morphospecies *X*, thus constituting the *FAD* of X (**Figure 2A**). For simplicity, I assume there are no fossils assignable to morphospecies *Y*. The age of *FAD X* represents a robust minimum estimate on the divergence time between the species *if* taxon *X* (including its fossils) has at least one morphological autapomorphy with respect to *Y*, as it implies that *Y* diverged from *X* before the first appearance of that autapomorphy (otherwise, we would expect *Y* to also have that character state) (**Figure 2A**).

However, if morphospecies *X* (including its fossils) has no morphological autapomorphies with respect to taxon *Y*, then *Y* could have budded off from lineage *X* at any time (**Figure 2B**) with the possibility that *FAD X* predates the emergence of lineage *Y*. In this case, the age of *FAD X* is not an unequivocal minimum estimate on the divergence time between the two lineages as it could either post-date of pre-date their time of divergence. Note, further, that character state reversals are commonly observed in morphological data, so there will be some probability that even if *X* has autapomorphies with respect to *Y*, which *Y* might still have budded off lineage *X*, having subsequently lost those characters (Wagner, 1998).

While the notion of budding has been part of paleontological reasoning for decades (e.g., see Raup, 1985) and underpins the FBD method of incorporating fossils (Heath et al., 2014), only recently have its implications for integrating neontological and paleontological data begun to be explored (Silvestro et al., 2018; Stadler et al., 2018). Below, I give two examples where the fact that the morphology of “hosts” of diverging DNA sequences might be subject to stasis can affect the way one interprets, and in the second case, calibrates DNA trees.

#### 2.1.1 The Dentist Who Infected Several Patients With HIV

**Figure 3:**
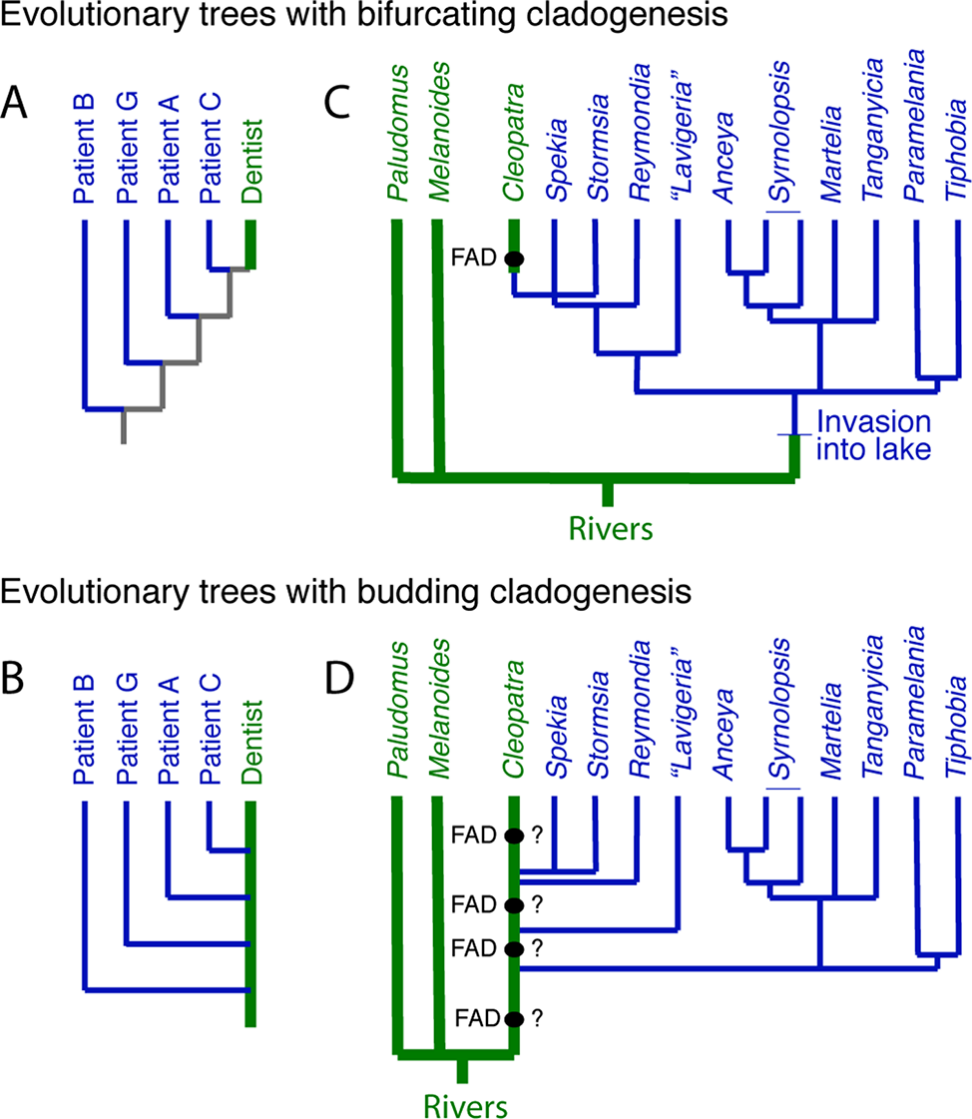
The distinction between bifurcating and budding cladogenesis matters for DNA trees. **(A)** Part of the evolutionary tree (*y* clone of the HIV-1 *env* V3 region) of the dentist and some of his HIV infected patients from Ou et al. (1992). The gray internal branches indicate that, without additional information, we do not know if the virus in the patients was derived from the dentist or not. **(B)** The tree from (A) redrawn with budding cladogenesis, making obvious the sequential infection of patients with HIV by the dentist. **(C)** Bifurcating tree at the genus level of three East African riverine and multiple endemic Lake Tanganyika gastropods, which suggests the riverine *Cleopatra* evolved from a lake endemic (Wilson et al., 2004)—the *FAD* of *Cleopatra* constrains the divergence of *Cleopatra* from its nearest relatives. **(D)** However, if the lineage that lead to the living *Cleopatra* invaded the lake several times (West and Michel, 2000), then the *FAD* of *Cleopatra* does not offer a reliable constraint on the divergences within the lake taxa, unless it can be shown to have synapomorphies with a subset of the lake endemics.

In a case that gained international notoriety, a DNA tree derived from a portion of the HIV genome verified that an HIV-positive dentist in Florida had accidently infected several of his patients (Ou et al., 1992). The DNA tree itself shows the dentist at the top of the tree (**Figure 3A**), which might suggest that the dentist acquired HIV recently from patient C, although without additional data, there is no way of knowing. However, once one recognizes that budding is possible, in the sense that the dentist remained unchanged as the host to the virus that was evolving within him, then a budding tree can be drawn (**Figure 3B**) where it is immediately obvious that the dentist sequentially infected several patients.

#### 2.1.2 Potential Example of Budding Cladogenesis—Multiple Invasions of Riverine Gastropods Into Lake Tanganyika?

The endemic thalassoid gastropods in Lake Tanganyika represent one of the many species flocks in the major East African lakes. Surprisingly, a widely distributed riverine and putative outgroup, *Cleopatra*, lies high in DNA trees of the group, buried deeply within the endemic Lake Tanganyikan clade (**Figure 3C**). A natural explanation for this topology is that *Cleopatra* is not an outgroup but had its origin in the lake to later invade the adjacent rivers (West and Michel, 2000; Wilson et al., 2004). Under this scenario, the oldest fossil morphologically assignable to *Cleopatra*, i.e., its *FAD*, might be used as a minimum age constraint on *Cleopatra*’s divergence from its closest relatives, the lake endemics *Stormsia* and *Spekia* (**Figure 3C**) or *Reymondia* (West and Michel, 2000).

However, another possibility, consistent with the DNA tree, is one of pervasive morphological budding cladogenesis (**Figure 3D**) (West and Michel, 2000). Under this scenario, the endemic lake fauna were derived from multiple invasions into the lake of populations of riverine snails that might have been morphologically indistinguishable from the living *Cleopatra* (or some allied forms [see Van Damme and Pickford, 2003]). In the bifurcating DNA tree, this would have left *Cleopatra* as sister group to the last lake lineage it gave rise to, as is observed. If this scenario is correct, then the oldest fossil *Cleopatra* will lack morphologic autapomorphes with respect to its lake descendent lineages, and thus, these fossils offer no minimum age constraint on the time of origin of the lake lineages.

This scenario has yet to be formally tested but highlights the fact that morphospecies-level interpretations of DNA-based topologies could be inaccurate if one ignores the possibility of morphological budding. It also highlights the importance of apomorphy-based minimum age constraints. Intriguingly, the budding scenario finds support in the fact that fossil *Cleopatra* are known to at least 12.5 million years ago (Van Damme and Pickford, 2003), older than the onset of rifting that led the formation of the lake ∼9–12 million years ago (Cohen et al., 1993).

### 2.2 Most Groups Have Problematic Potential FADs

Given that the number of diagnostic features drops as one approaches the origin of a group, and given that most fossils are morphologically incomplete, most groups have problematic fossils that might conceivably be *FAD*s, but where there is insufficient morphology preserved to be sure. If one is simply trying to establish reliable brackets on divergence times, then the best practice is to only use morphologically secure FADs, which are typically younger than older potential *FAD*s (Donoghue and Benton, 2007). This approach also ameliorates to some degree the sensitivity of temporal calibrations to the phylogenetic uncertainty in the placement of key fossils.

### 2.3 Dating *FAD*s

#### 2.3.1 The Basis for the Dating of *FAD*s Needs to Be Explicit

Parham et al. (2012) deal with the need for explicit statements about how the absolute age constraints on an *FAD* have been established, including the locality and stratigraphic level the specimen(s) came from, and the basis of the absolute time assigned to that stratigraphic level. Here, for those not familiar with how ages are assigned to fossils, is the reason for their insistence.

#### 2.3.2 Only the Youngest Fossils can be Dated Directly

There are two standard ways of directly dating fossil material *via* radioisotopes. The first and more versatile is ^14^C dating, but its half life is so short (5,730 years) that reliable dates can only be obtained for fossils up to about 40,000–60,000 years old (Taylor and Bar-Yosef, 2014). The second is uranium series disequilibrium dating of carbonates (which biologically includes corals) including ^238^U/^234^U/^230^Th and ^235^U /^231^Pa datings (Edwards et al., 2003). But it also can only be applied to very young fossils, just over 600,000 years (for ^230^Th dating, see Stirling et al. (2001)).

#### 2.3.3 The Dating of the Vast Majority of *FAD*s Is Indirect

Ultimately, all absolute dates in the rock record are derived from radiometric dates. These typically provide an estimate of when the minerals that contain a relevant radioisotope crystallized out of molten rock, either in a magma chamber (most commonly zircons, which trap ^235^U and ^238^U) or as a volcanic ash is erupted from a volcano (most commonly sanidine feldspar, which traps ^40^Ar). In an ideal case, a key fossil will lie in sediments that are bracketed by younger and older dateable volcanic ash layers. Even better is the rare case where a fossil is actually embedded in a datable rock—for example, the rhinocerotid skull found in a 9.2 million year old ignimbrite flow erupted from a volcano in Turkey (Antoine et al., 2012). The worst case scenario is where the fossil of interest lies in sediments with no nearby igneous rocks, and without fossils that can be temporally correlated with similar fossils elsewhere (i.e., using biostratigraphy) where constraining radiometric dates are available. Thus—for example, this is the case for the famous Ediacaran localities of enigmatic latest pre-Cambrian fossils in the Ediacara Hills in South Australia that have yet to be dated with any sort of precision.

The more normal situation is where local radiometric dates (or other well dated events, such as switches in the Earth’s magnetic polarity) are not available, but where biostratigraphy can be used to correlate with places that have some age control. The dating of the famous Cambrian Burgess Shale fauna is one of these—its age assignment is based on biostratigraphy on the assumption that its trilobite fauna, specifically *Ehmaniella*, lived at about the same time at other localities where radiometric dates are available (see p.441 in Peng et al., 2012). However, while the order in which species appear and disappear in the fossil record is pretty consistent in different geographic areas, species typically take time to reach their maximum geographic range and are often extirpated (become locally extinct) heterogeneously on their way to extinction (Foote, 2007; Foote et al., 2007; Liow and Stenseth, 2007)—thus, paleontologists assume that the presence of a species in two geographic areas only indicates approximately the same point in geologic time (see Figure 7.9 in Taylor, 1987, reproduced in Donoghue and Benton, 2007).

In absolute terms, spatial asynchrony in times of first and last appearances of a species is typically less than an average species duration [∼2 million years for Cenozoic mammals, for example (Marshall, 2017)], perhaps no larger than a few hundred thousand years, but conservatively ± 1 million years. Radiometric dating errors are typically less than 1% of the age of the rock (Cohen et al. 2013) but can be as low as 0.1% for Ar-40/Ar-39 dating (Sprain et al., 2019) and down to almost 0.01% for U-Pb dating (Burgess et al., 2014), which is probably smaller than the uncertainty in pre-eruptive residence time of zircons in magma chambers (where zircons form before they are erupted). However, in some cases, the age uncertainties can be large—for example, the Dominican amber is very poorly constrained with an age range from 15 to 20 million years ago (Iturralde-Vinent and MacPhee, 1996; Ramírez et al., 2007).

Finally, there is variety of other indirect methods available for dating fossils. For example, the ratio of ^87^Sr/^86^Sr can be used to estimate the age of deposition of sediments, or hard tissues such as the shells of fossil brachiopods, as long as one knows roughly how old the fossil is [in some cases, this prior knowledge can be very imprecise—for example, for younger fossils, it is often sufficient to simply know that it is Cenozoic in age to make use of the approach (McArthur et al., 2012)]. The precision can be as high as ± 0.1 million years (McArthur et al., 2012).

#### 2.3.4 Dating Uncertainties Are Typically Relatively Small

Generally speaking, if good dates are available for *FAD*s (see Benton and Donoghue (2007); Clarke et al. (2011); and especially Benton et al. (2015) for many examples across the tree of life), the dating errors are small compared with the approximate divergence time, in the order of a million years, typically shorter than a species duration. However, sometimes, the dating of key *FAD*s is imprecise—for example, the age of the oldest fossil hominin, *Sahelanthropus*, lacks precision, somewhere between 6.5 and 7.5 million years old [see discussion in Benton and Donoghue (2007) and Reis et al. (2018)], an uncertainty that amounts to ∼14% the total age of the fossil.

## 3 Maximum Age Constraints—Step 1: Estimating the Size of ΔT_Gap_

While establishing robust minimum age constraints is relatively straightforward (using well-dated, well-diagnosed apomorphy-rich fossils), there are no well established procedures for establishing robust maximum age constraints, a challenge that has plagued node-dating approaches and has led some to favor alternative approaches (for discussion, see Donoghue and Yang (2016)). In this section, I deal with constraining the time of origin of the oldest fossilizable apomorphy, which is estimating the size of ΔT_Gap_ (**Figure 1A**). In the following section (Section 4), I then deal with estimating the size of the gap between the true time of origin of the oldest fossilizable apomorphy and the actual divergence time (ΔT_Div-1stApo_). Note that total evidence approaches (Pyron, 2011; Ronquist et al., 2012), by ducking the need to estimate maximum age constraints, simply ignore the fact that fossil age estimates of divergence times are too young.

Below, I discuss five approaches to estimating the size of ΔT_Gap_: 1) *confidence interval* approaches, which use quantitative measures of the richness of the fossil record of individual lineages within the focal taxon to estimate how much of the fossil record might be missing; 2) the *taphonomic control group* approach which uses the ages of non-focal-taxon fossils that are older than the focal taxon’s *FAD* to provide evidence that the absence of the focal taxon is real; 3) the *super-taxon* approach which uses an un-calibrated timetree to combine all the *FAD*s across into a “super-taxon,” which is then analyzed using a confidence interval approach; 4) *clade diversity dynamics* approaches that model the stratigraphic ranges of species not preserved in the fossil record; and 5) *FADs of successive outgroups* approach, which can be used when the fossil record is quite rich. The first two methods can be applied to multiple lineages with the focal clade, or to entire clades. The latter three were designed for the analysis of entire clades (**Table 1**).

**Table 1:** Methods that use fossil data to place soft maxima on times of origin of first fossilizable apomorphies, that is, to estimate the size of ΔT_Gap_ (Figure 1A). See text for citations.

Method	Can be applied to:		Ancillary data needed
	Multiple lineages in focal clade	Whole clade	
1. Confidence intervals	Yes	Yes	Fossil record richness within focal clade
2. Taphonomic control groups	Yes	Yes	Fossil record outside of focal clade
3. Super-taxon confidence intervals	No	Yes	Un-calibrated ultrametric tree; multiple *FAD*s
4. Clade diversity dynamics	No	Yes	Extant and fossil species richness
5. *FAD*s of successive outgroups	No	Yes	*FAD*s of successive outgroups

Note that all these methods place soft maxima on the target time of origin (Marshall, 1990a, Marshall, 1990b; Yang and Rannala, 2006; Benton and Donoghue,2007; Donoghue and Benton, 2007), that is, they provide a maximum age constraint at some level of confidence or probability, typically, 95%.

It has also been suggested that ancestral fossils can also be used as hard maxima (e.g., see Marshall, 1990b). But even if a taxon has a morphology consistent with being ancestral to some target species, and is older than the target species as it should be (Smith, 1994, chapter 6), unless the proportion of all species preserved is very high (see Section 4.1.1 for some examples), the chances are that the putative ancestral fossil will be sister to the focal taxon not ancestral (Foote, 1996) and thus is unlikely to represent a valid maximum constraint. The reason is simply that, for any given taxon, the proportion of ancestral lineages is small compared with the number of older non-ancestral lineages (Foote, 1996).

### 3.1 Method 1—Confidence Intervals to Constrain ΔT_Gap_

This approach uses the density of fossil finds through time within the focal taxon to quantify **Δ**T_Gap_.

#### 3.1.1 Simplest Approach

Intuitively, the richer the fossil record the closer the *FAD* will be to the true time of origin of the first fossilizable apomorphy of the group (T_1stApo_) (Strauss and Sadler, 1989; Marshall, 1990a; Marshall, 1990b). For an extant taxon known from *n* distinct fossil localities, the sizes of the temporal gaps between successive localities will be exponentially distributed if fossilization and fossil recovery were random, and the confidence interval T_C_ on T_1stApo_ at a confidence level, *C*, is given by

(2) $${\text{T}}_{\text{C}}=FAD{\left(1-C\right)}^{-1\text{/}n}$$

The unbiased estimate of the T_1stApo_ is the average gap size, *FAD*/*n*. In the paleontological literature, equation (2) has an exponent of −1/(*n*−1), not −1/*n* (see equation [8]), reflecting the fact that the equation was developed for extinct species where one needs to condition on the last fossil occurrence, which also results in the gaps sizes between successive localities having a Dirichlet rather than exponential distribution (Strauss and Sadler, 1989).

#### 3.1.2 Likelihood Formulation of the Simplest Approach

**Figure 4:**
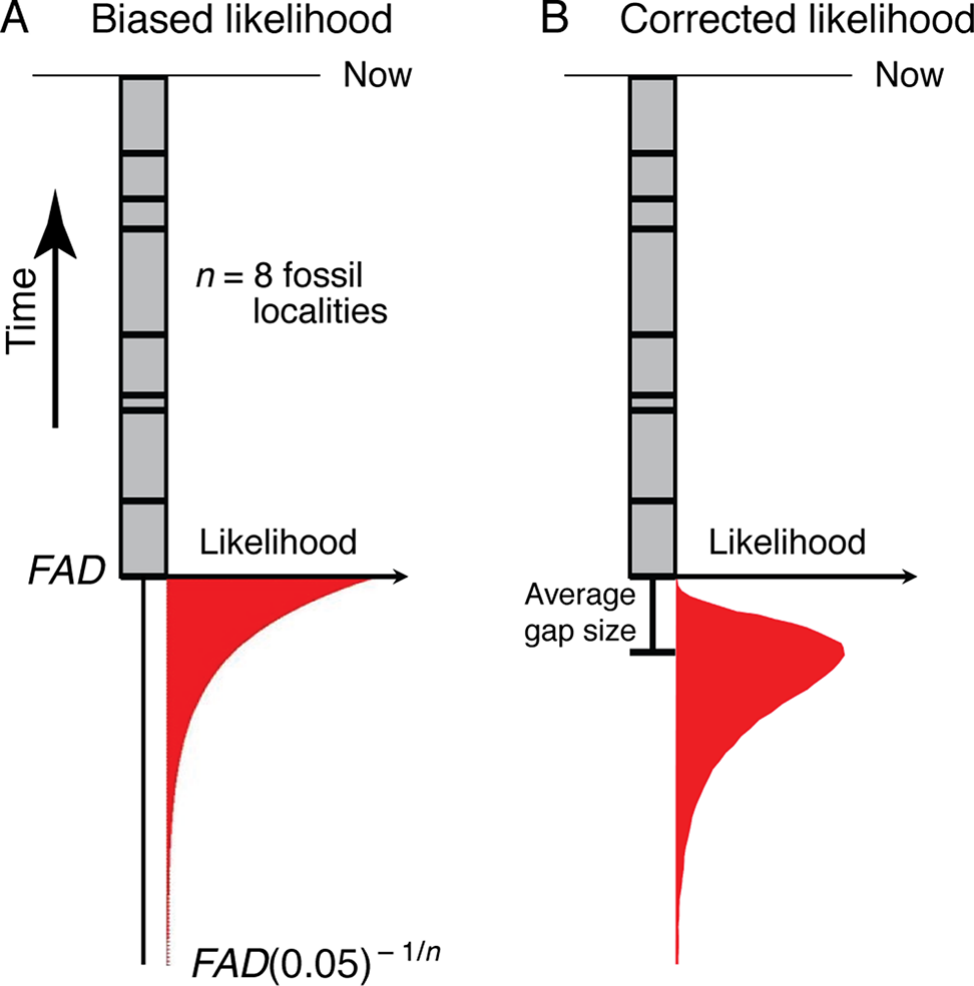
**(A)** The exponential likelihood density function for how much older the true time of origin of a morphospecies is than its *FAD* (ΔT_Gap_) under the assumption of random fossilization. **(B)** The likelihood density function in (A) corrected for a finite sample size (see Section 3.1.2).

Bayesian approaches for generating timetrees typically require specification of priors on the times of origin, which need to be expressed as likelihoods. Thus, the frequentist formulation described above needs to be translated into a likelihood framework. This has been done by Strauss and Sadler (1989)—the fact that stratigraphic gap sizes will be distributed exponentially under random fossilization (Strauss and Sadler, 1989) suggests that the most appropriate prior will be exponentially distributed (**Figure 4A**). However, maximum likelihood analysis of monotonic distributions such as the exponential are biased, with the maximum likelihood estimate corresponding to a zero temporal range extension (Strauss and Sadler, 1989)—the maximum likelihood estimate for the time of origin of a clade (T_ML_) is the age of its oldest known fossil (**Figure 4A**):

(3) $$T_{\text{ML}}=FAD$$

However, a correction for the maximum likelihood estimate for the time of origin, T*_ML_, given a finite sample size (*n*), can be found by multiplying T_ML_ by (*n* + 1)/*n*:

(4) $$\begin{array}{l} \text{T}{*}_{\text{ML}}=FAD(\text{n}+1)/n\\ \;=FAD+FAD/n \end{array}$$

(5) = FAD + average temporal gap between fossil localities

Thus, as for the frequentist analysis above, the most likely time of origin is the average gap size below the *FAD*.

##### 3.1.2.1 The Paleontologically Most Appropriate Prior

This analysis suggests that the paleontologically most appropriate prior on the time of origin of a clade, at least that can be detected with the fossil record, will have a mode that extends an average gap size below the oldest fossil, and, using equation (2), a 95% tail extending ∼*FAD*(0.05 )^–1/^ *^n^* beyond the age of the oldest fossil locality (the *FAD*) (**Figure 4B**). Of the priors currently available for the construction of timetrees, the lognormal distribution has this shape (Ho and Phillips, 2009), as do the gamma (Yang and Rannala, 2006) and truncated Cauchy (Inoue et al. 2010) distributions depending on how they are parameterized. Given a *FAD* and the number of distinct localities (*n*) for a taxon, the mean and variance of the corresponding lognormal prior can be found (see Appendix A for derivation):

(6) $$\mu =\ln\left(FAD\right)-\ln\left(n\right)+\sigma^{2}$$

(7) $$\sigma^{2}={\left(-0.8224+0.5\sqrt{2.7055-4\left(-2.9957/n-\text{ln}\left[n\right]\right)}\right)}^{2}$$

##### 3.1.2.2 Comparison With an FBD Process Timetree

**Figure 5:**
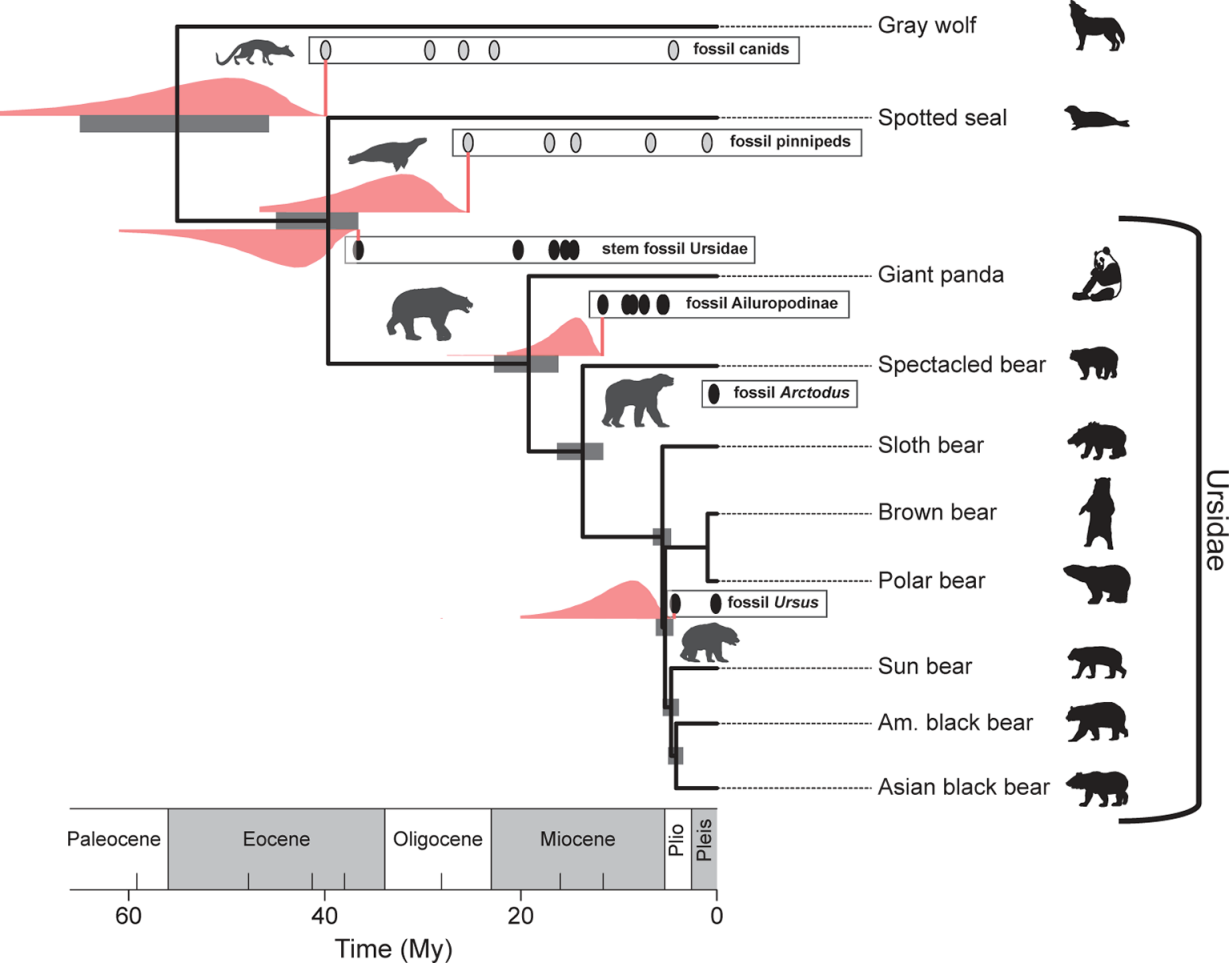
Broad agreement in the estimated uncertainties in the divergence times of selected bear and outgroup lineages based on the FBD (fossilized birth–death) process (gray bars) (Heath et al., 2014) and those based on lognormal-like likelihood analysis of the fossil records depicted (pink distributions; see also Figure 4). Pink vertical lines correspond to the *FAD*s. The pink range extensions have been added to a reproduction of Figure 4 from Heath et al. (2014).

Interestingly, Heath et al. (2014) analysis of extant bear divergence times, using their “fossilized birth–death” (FBD) process for constructing timetrees, provides uncertainty estimates on their divergence times that are broadly congruent with priors developed with the procedure outlined above (see **Figure 5**). This is perhaps not surprising given that the same fossil data were used in both the computation of the confidence intervals and in the FBD analysis, which takes into account the average preservation rate based on the fossils incorporated into the analysis. Nonetheless, it is heartening that there is broad agreement between a purely paleontological approach (the paleontological parameterization of the lognormal distribution) and an approach that incorporates fossil and DNA data, as well as an explicit branching model as part of its inference engine (the FBD process). As a methodological aside, note that modifications of the FBD model can accommodate variation in diversification and fossil recovery rates [e.g., see Gavryushkina et al. (2014)].

#### 3.1.3 Difficulties With the Simplest Approach

Generally speaking, we do not expect the probability of finding fossils to be stochastically constant through a lineage’s temporal range; instead, we expect the probability of finding fossils to decrease the closer we approach the time of origin (**Figure 1B**) [see Marjanović and Laurin (2008) for an empirical example]: 1) the number of separate lineages will approach one, the initiating lineage; 2) the geographic range is likely to be smaller; and, 3) there will be progressively less apomorphies of the group, making it progressively harder to diagnose incomplete fossils and thus unequivocally assign taxa to the clade of interest. Moreover, (4) the rock and fossil records are spotty both temporally and geographically [e.g., see Wagner and Marcot (2013) and Marjanović and Laurin (2008) for some empirical examples], which adds further uncertainty, although we now know a great deal about the controls and therefore the structure of the sedimentary rock record (Patzkowsky and Holland, 2012; Holland, 2016). Below is a simple example that illustrates the impact of the incompleteness of the rock record has in computing confidence intervals, and then I discuss ways in which the decrease in preservation potential can be accommodated.

##### 3.1.3.1 Example—Trying to Date the Time of Origin of a Sand Dollar

As part of my Ph.D., I attempted to quantify the uncertainty in the divergence time between several sand dollar species. Multiple confounding difficulties made this difficult, which are exemplified here by my analysis of the time of origin of the genus *Mellita*.

**Figure 6:**
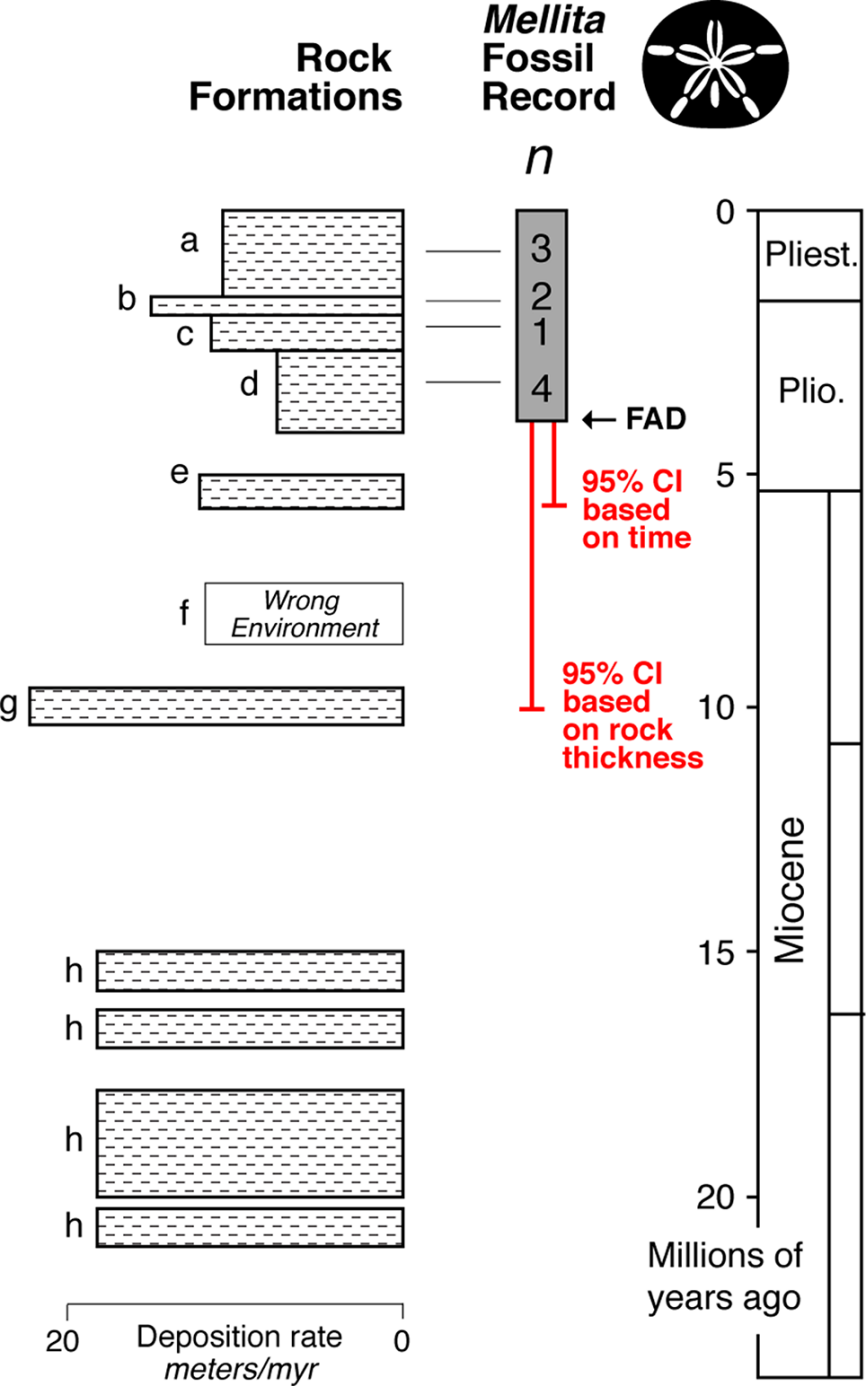
Idealized representation of the rock formations on the Atlantic Coastal Plain region of the USA (Lindberg, 1983), with the fossil record and minimum number of localities (*n*) for each formation for the fossil sand dollar *Mellita* (silhouette). The *FAD* in this region is ∼4 million years old, with the end of the 95% confidence interval (equation 1) extending to ∼5 million years ago. However, when one takes into account the substantial gaps in the rock record, and the fact that some rock formations were deposited in environments inimical to *Mellita* (formation f), the 95% confidence interval extends to ∼10 million years.

The fossil record of *Mellita* is relatively strong with at least 10 localities alone from the Atlantic Coastal Plain of the USA (Lindberg, 1983) (**Figure 6**). The *FAD* in this region is ∼ 4 million years old, and the 95% confidence interval extends to about ∼5 million years old. However, while the rock record always appears complete in outcrop, it is typically riddled with temporal gaps (Sadler, 1981; Holland, 1995; Patzkowsky and Holland, 2012; Holland, 2016). Further, even when rocks are present in a given time interval, they might not represent suitable environment for the taxa of interest. For example, *Mellita* only lives on sandy substrates, and thus, its fossils are only found in sandstones—the St. Mary’s Formation (formation “f” in **Figure 6**) is a muddy unit, and so, we don’t expect fossil *Mellita* to be found there. When confidence intervals are calculated by rock thickness of the sandstone formations, a way of taking into account the major temporal gaps in appropriate deposition, the 95% confidence interval extends into the Choptank Formation (formation “g” in **Figure 6**), about ∼10 million years in age, doubling the soft maximum estimate.

Moreover, *Mellita* and its sister genus *Leodia* have current geographic ranges that extend to Uruguay (Mooi and Peterson, 2000; Martínez and Mooi, 2005), and the now-extinct basal members of the clade to which *Mellita* and *Leodia* belong are only known from Uruguay, Argentina, and Chile (Mooi et al. 2000). But there are no fossil *Mellita* or *Leodia* known south of Caribbean, and so, it is quite possible that these genera had their origins in geographic region from which there has been very little paleontological effort exerted, the Atlantic coast of South America; the fossil record might be giving us a record of when *Mellita* and *Leodia* migrated into Caribbean (and then into the Pacific), not when they originated. Using the fossil record of these now-extinct basal members of the clade as taphonomic controls (see Section 3.2), a soft maximum limit on the time of origin of the genus *Mellita*, is the Middle to Upper Miocene boundary (Mooi et al., 2000), 11.6 million years ago.

Relatively few analyses of this kind have been undertaken [although see Marjanović and Laurin (2008)], but it exemplifies how the temporal and geographic spottiness of the rock and fossils record, and our spotty knowledge of them, adds considerable uncertainty to when a taxon originated.

##### 3.1.3.2 Dealing With a Non-Random Fossil Record

In **Figure 1**, I have emphasized the fact that the probability of fossil recovery generally drops the further back we go in the history of a clade. The importance of accommodating this is illustrated *via* a thought experiment—if one assumes that crown group birds existed in the Cretaceous, but at, say, 1/10^th^ Cenozoic preservation rates, the 95% confidence on the crown group time of origin of a relatively fossiliferous group, the Caprimulgiformes, increases to ∼90 million years from the ∼70 million year estimate under the assumption of random fossilization (Marshall, 1999).

Marshall (1997) developed a method for accommodating decreasing probabilities of fossil recovery with time (or in fact any non-random distribution of fossil recovery potential). However, we do not yet have standard methods for developing the required empirical non-random fossilization potential curves (Marshall, 2010). Nonetheless, Marjanović and Laurin (2008) provide an example of a compound model that used sedimentary rock outcrop area through time coupled with an exponential model of diversification punctuated by mass extinctions to model the diversity trajectory of the living amphibians (the Lissamphibia). They parameterized (and tested the efficacy of) their model with the Lissamphibian fossil record (1,207 localities across the global history of the group) to establish confidence intervals on its time of the origin using the generalized confidence interval approach of Marshall (1997). Marjanović and Laurin’s (2008) study may serve a good model for realistic fossil-based confidence intervals for higher taxa, and thus also for establishing priors on divergence times.

There are also simpler analytic methods for accommodating trends of decreasing fossil recovery within the known stratigraphic range to approximate the assumed further decrease in fossil recovery beyond the known stratigraphic range. The first methods used the Weibull distribution, which assumes a decreasing rate of preservation (Roberts and Solow, 2003; Solow and Roberts, 2003). However, these methods tend to overestimate the true temporal endpoint (Rivadeneira et al., 2009). The most recent and best performing method is the flexible beta method of Wang et al. (2016), but all of these methods assume simple monotonic change in fossil recovery potential, unlike the empirically richer (and demanding) approach of Marjanović and Laurin (2008).

##### 3.1.3.3 Another Way of Ameliorating the Difficulties—the Origin of Hominins

Another way of trying to ameliorate the difficulties associated with the decreasing probably of encountering fossils the further back we go in time (**Figure 1B**) is to work only with the oldest part of a lineage’s fossil record where the fossil recovery rate is likely to have been relatively constant (however that is determined). Thus—for example, from the divergence of our own species from chimpanzees to about 4 million years ago, our own evolutionary branch may well have consisted of just one lineage. Fossils come from just *n* = 4 fossiliferous places in Africa for this early part of our history, one for *Sahelanthropus* [which provides our lineage’s *FAD* of between 7.5 and 6.5 million years ago; see Benton and Donoghue (2007); Reis et al. (2018) for discussion of the age uncertainty], one for *Orrorin* [which has yielded fossils from ∼6.0 to 5.7 million years ago (Sawada et al. 2006)] and two for *Ardipithecus* (∼5.8–5.2 (Haile-Selassie 2001) and ∼4.4 million years ago (White et al. 2009)]. This yields a stratigraphic range (*R*) of 2.1–3.1 million years. For paleontological data, the confidence interval T_C_ on the *FAD* at a confidence level, *C*, is given by (Marshall, 1990a):

(8) $${\text{T}}_{\text{C}}=R[{\left(1-C\right]}^{\left(-1/n-1\right)}-1]+FAD$$

The 95% confidence interval on this part of the hominin fossil record using equation (8) extends to between 12.8 and 10.1 million years ago, with an unbiased estimate (the average gap size added to the *FAD*) of between 8.5 and 7.2 million years ago. This is in good agreement with the taphonomic control group approach (see Section 3.2 immediately below), where there are several fossil localities that yield hominin fossils at about 10 million years ago with no evidence of fossils assignable to either the chimpanzee or hominin lineages (Benton and Donoghue, 2007; Reis et al., 2018).

### 3.2 Method 2—Constraining ΔT_Gap_ With Taphonomic Control Groups

The difficulty in quantifying the fossil recovery potential of a taxon beyond its known stratigraphic range has led many to rely on a more qualitative approach, the age of taphonomic control groups found beyond the *FAD* of the focal taxon as a maximum estimate for the time of origin. Taphonomy is the study of how organisms decay and become fossilized (Behrensmeyer et al., 2000), and taphonomic control groups are groups that are frequently found preserved in the same rocks, or at least the same environments, as the focal taxon—thus, their preservation in rocks older than the *FAD* of the focal taxon is taken as an indication that the focal taxon had not yet evolved (Bottjer and Jablonski, 1988; Marshall 1990b; Benton and Donoghue, 2007; Donoghue and Benton, 2007). Of course, the co-occurrence of taxa is typically never 100%, and so the first appearance of the taphonomic control group, *per se*, may not be a fully robust maximum bound on the time of origin of the focal taxon, a supposition that finds some empirical support (Clarke et al. 2011). To control for geographic incompleteness (see section 3.1.3.1 above), the control group must be found in the same broad geographic region where we think the focal group originated.

#### 3.2.1 Example—The Origin of the Sand Dollar *Clypeaster*

The clypeasteroid echinoids, the sand dollars, and sea biscuits (e.g., *Clypeaster*) have a relatively rich fossil record. Ali (1983) documents 397 species in the fossil record known from 768 localities (so on average, each species is known from about two localities). With this quality of fossil record, we have reasonable confidence that the genus had its origin in the equatorial Tethys Sea (**Table 2**), now seen in the rock record around the Mediterranean and in the Middle East. The oldest fossils are in Middle Eocene. Other irregular echinoids are found in the region in the Lower Eocene and in the older Paleocene [see Souto et al. (2019) and also the Paleobiology Database (PBDB), although for most groups the PBDB only documents a portion of the known fossil record (Marshall et al., 2018)]. Thus, a reasonable maximum estimate for the time of origin of *Clypeaster* was by beginning of the Eocene, and we can be even more certain that it had its time of origin somewhere in the interval bracketed by its Middle Eocene *FAD* and the beginning of the Paleocene.

**Table 2:** Species occurrences of the 397 fossil species in Ali’s (1983) compilation of the genus *Clypeaster* by time and geographic region.

Duration (myr)	Epoch	E. Pacific	Caribbean	Tethys Ocean (Mediterranean)	Indian Ocean	West Pacific
2.6	Pleistocene	3	6		10	4
2.8	Pliocene	10	14	16	20	10
17.7	Miocene	7	44	508	31	3
10.9	Oligocene	1	35	23	10	1
7.3	Upper Eocene		2	6	2	
6.6	Middle Eocene			2		
8.2	Lower Eocene					

The intensity of the shading is proportional to the log(# occurrences/myr). The rich fossil record of *Clypeaster* indicates that the genus originated in the region of what is now the Mediterranean Sea, which was the Tethys Ocean at the time of *Clypeaster*’s FAD.

### 3.3 Method 3—The Super-Taxon Confidence Interval Approach

**Figure 7:**
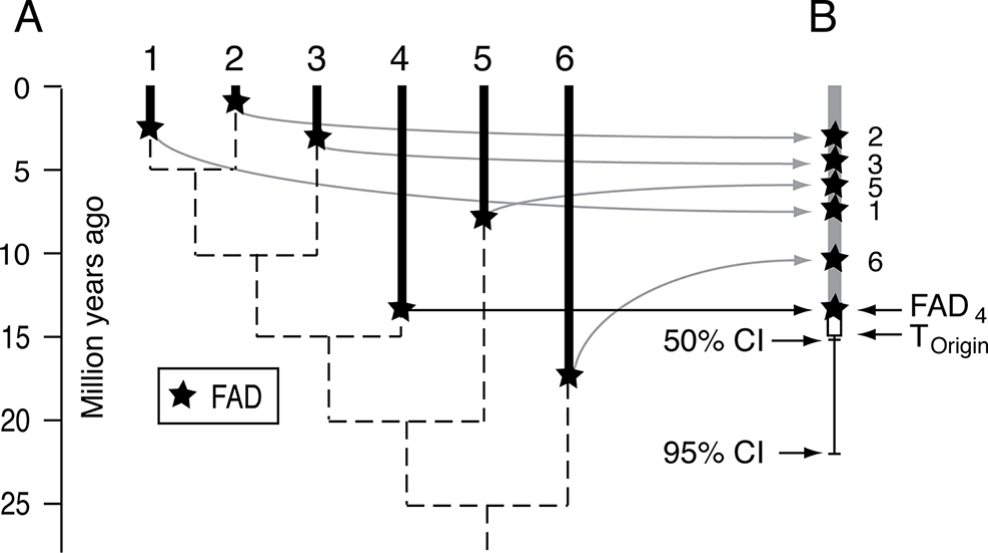
Schematic for the super-taxon approach for using multiple *FAD*s to constrain the time of origin of a clade. **(A)** Hypothetical ultrametric tree (dashed lines) with the *FAD*s for each lineage. **(B)** The method uses the branch lengths of the ultrametric tree to map the *FAD*s onto a single lineage, or super-taxon, and then uses confidence intervals (equation [1]) to bracket the time of origin, that is estimate the size of ΔT_Gap_ for the entire clade. See Marshall (2008) for further explanation. Adapted from **Figure 1** in Marshall (2008).

Confidence intervals on stratigraphic ranges have long been used to assess likely times of extinctions, especially mass extinctions (Marshall, 1995a; Marshall and Ward, 1996; Jin et al., 2000; Marshall, 2010; Wang and Marshall, 2016). The most powerful approach for mass extinction victims is to combine all the data, effectively collapsing all the species into a single super-taxon (Wang et al., 2009). The same logic has been applied in reverse, using all the *FAD*s to form a super-taxon, to estimate the time of origin of a clade, as well as the internal branches, where the relative positions of the *FAD*s are adjusted by the relative length of their branches on an un-calibrated ultrametric tree (timetree) (Marshall, 2008)(**Figure 7**). This approach has the advantage of not requiring estimates of maximum divergence times but has the disadvantage of the potentially unrealistic assumptions about the fossilization process (although see Marshall (2008) for discussion). It differs from most approaches for constructing time trees in that it is sequential in nature—an ultrametric tree is constructed first in the absence of any absolute time constraints, and then the scaling of that tree is established using the super-taxon paleontological approach.

#### 3.3.1 Congruence Between the Taphonomic Control Group and Super Taxon Methods?

Several analyses of turtle divergence times (Joyce et al., 2013; Pereira et al., 2017; Shaffer et al., 2017) have employed the best practices for establishing *FAD*s and used taphonomic control groups for establishing soft maxima (Joyce et al., 2013) (**Table 3**). All three studies used the same fossil calibrations, updated from (Near et al., 2005), except for Pereira et al. (2017) who used a updated minimum paleontological date for the root node. Marshall (2008) also used the Near et al. (2005) data and the super-taxon approach to estimate turtle divergence times. When the super-taxon approach is adjusted by eliminating the three *FAD*s identified as being questionable by Joyce et al. (2013) [*‘‘Aspideretes’’ maortuensi* (calibration lineage 6), *Proterochersis robusta* (calibration lineage 1), and *Santanachelys gaffneyi* (calibration lineage 5), which Marshall’s method also indicated as being problematic], leaving lineage 10 (*Baltemys*) as the calibration lineage, the new super-taxon results are broadly congruent with the taphonomic control group studies (**Table 3**).

**Table 3:** Taphonomic control group and super-taxon confidence interval approaches to estimating maximum (and minimum) age constraints on turtle divergence times are broadly congruent when they all use the same fossil *FAD*s.

Study	Method for assigning maxima	Turtle crown group age (million years ago)	
		Mean	95% HPD/confidence
Joyce et al. (2013)	Taphonomic control group	212	195–231
Pereira et al. (2017)	Taphonomic control group^1^	199.5	179–225
Shaffer et al. (2017)	Taphonomic control group^1^	220	194–251
Marshall (2008) ^2^: H-bar = 1	Super-taxon CI	220	209–259
Marshall (2008) ^2^: H-bar = 2	Super-taxon CI	214	209–232

^1^Following Joyce et al. (2013).

^2^With problematic FADs eliminated (see text).

### 3.4 Method 4—Using *FAD*s of Successively More Inclusive Clades

As one examines successively older rocks focal taxa disappear with only successively more plesiomorphic sister groups being found (from the point of view of the focal group). Thus, using a taphonomic control group type reasoning, the presence of these plesiomorphic taxa without taxa from the focal group gives the sense that the focal group had not yet evolved, providing a maximum age estimate for the focal taxon. Following this logic—for example, Gustafsson et al. (2010) used the *FAD* of the entire monocot clade of plants as a maximum age constraint on the time of origin of the orchids within the monocots.

This approach has been formalized for cases where the order in which a series of clades appear on a cladogram is matched by the temporal order of those clades’ *FAD*s (Hedman, 2010). The method is developed in a Bayesian framework and is implemented in R (Lloyd et al., 2016). It can been adjusted for groups that violate this requirement by leaving out inconsistent groups (Friedman and Brazeau, 2011; Friedman et al., 2013) and has the virtue that it does not require any estimate of preservation and fossil recovery rates.

The method has been applied to the origin of digit bearing tetrapods, with a 95% credible interval from∼396 to 427 million years ago (Friedman and Brazeau, 2011), although this example highlights the potential discrepancies between times of origin of fossilizable apomorphies (digits in this case) and lineage divergence times—the relatively rich fossil record of the first tetrapods and their precursors indicates that the time of origin of digit bearing tetrapods considerably post-dates the time of divergence of tetrapods from the nearest living relatives, the lungfish or coelacanths (Marshall and Schultze, 1992; Min and Schultze, 2001).

### 3.5 Method 5—Modeling the Stratigraphic Ranges of Missing Species

Typically, only a small proportion of all species that have ever existed are found in the fossil record. For example, less than 7% of all living primate species are found as fossils (Martin, 1993; Tavaré et al., 2002; Wilkinson et al., 2011). Martin (1993) noted that when the proportion of species preserved is small, and especially for clades that have been steadily expanding, the oldest fossil species found in the group might be several species durations younger than the very first species. For example, he simulated the expansion of clade over 16 species durations from one to 48 extant species, which resulted in a total of ∼380 species, ∼330 of which were extinct. A random sample of 3% of the extinct species yielded on average an oldest fossil that was five species durations younger than the base of the clade—his fossil record was missing about the first 30% of the true stratigraphic range of the group.

This approach was formalized by Tavaré et al. (2002) and applied to the crown group primate fossil record where the oldest fossil is ∼55 million years old (that is, we ignored the extinct stem-group primates, the plesiadapiformes). This paleontological method yielded a mean estimate of 81.5 million years for crown group primate origins, a date compatible with the molecular clock estimates of the divergence of primates from their nearest relatives [which in 2002 was about ∼90 million years ago (Tavaré et al., 2002)]. More recently, Wilkinson et al. (2011) developed an extension of Tavaré et al.’s (2002) approach for integrating paleontological and molecular data, obtaining a very similar result, with a mean estimate of 84.5 million years ago for crown group primates.

Foote et al. (1999) employed a different analytic approach but also used a branching process as well as explicit preservation rates to determine how deep into the Cretaceous several mammalian orders likely extended. Foote et al. (1999) found that the fossil preservation rates for the better preserved mammalian orders give much younger times of divergence, much closer to the end of the Cretaceous, which is dated to 66 million years ago, in conflict with the older crown group primate date.

The reason for the discrepancy has not been determined, but while it is not unreasonable that a relatively poorly preserved group of mammals, crown group primates, for example, might have a very deep time of origin; it is harder to believe that all the other better preserved mammalian orders, which all diverged from each other at about the same time as primates, also had a similarly deep time of origin. Note, however, that Wilkinson et al. (2011) 95% confidence interval on the time of origin of crown primates ranges from 69.2 to 103.5 million years ago, its upper limit compatible with Foote et al.’s (1999) analysis.

Primates continue to be a test case for combined DNA-paleontological timetree construction. For example, Reis et al. (2018) find that crown group primates originated toward the younger end of the range established by Wilkinson et al. (2011), somewhere between 79.2 and 70.0 million years ago for their preferred analysis (using autocorrelated rates of molecular evolution), or perhaps 71.4–63.9 million years ago (with uncorrelated rates). They also find that primary sources of uncertainty in the analysis are associated with fossil calibration uncertainty.

Tavaré et al. (2002) approach assumed logistic diversification, conditioned on the number of living species, the oldest fossil crown group primates, and the richness of the crown group primate fossil record. Newer paleontological approaches have been developed to estimate the amount of time missing history prior to *FAD*s that explicitly use the fossil record to calculate average speciation, extinction, and preservation rates from the fossil record (Bapst, 2013; Nowak et al., 2013). Most recently, Wagner (2019) has developed a method for estimating branch durations and stratigraphic gaps in phylogenies when rates of speciation, extinction, and fossil sampling vary with time.

### 3.6 Multiple Calibration Points and Cross Validation

The first two methods (confidence intervals and taphonomic control groups) make use of multiple calibrations across the tree (**Table 1**). This has the advantage that the process of calibration is not so dependent on difficulties that might be associated with any one specific lineage (e.g., see the discussion of the sand dollar *Mellita* for an example of these difficulties, section 3.1.3.1).

The use of multiple calibrations also allows for the possibility of cross validation, that is, the search for consistency between the temporal calibrations of one calibration to the next.

#### 3.6.1 Cross Validation in Light of the Bias in the Fossil Record

The initial idea of cross validation was to see if various subsets of calibration points (*FAD*s) yielded similar absolute ages for the timetree, with the goal of eliminating calibrations that yielded anomalously young or old divergence time estimates (Near and Sanderson, 2004; Near et al., 2005). However, given that *FAD*s all underestimate the divergence times they are being used to estimate, simple cross-validation on *FAD*s will provide divergence time estimates that are too young, eliminating the best calibrations (Marshall, 2008), as well as the worst. To overcome this shortfall, rather than cross-validating on the *FAD*s, cross-validation may be performed using the temporal ranges between the minima (*FAD*s) and soft maxima (Clarke et al., 2011), however those soft maxima are established. However, cross-validation is not generally recommended because the sequential use of single calibrations does not have the same effect as the simultaneous use of all the calibrations (Warnock et al., 2015).

## 4 How Much Older Are Divergence Times Than the True Times of Origin of the First Fossilizable Apomorphies—How Big is ΔT_Div-1stApo_?

So far, we have ignored the second step in the estimation of maximum age constraints on divergence times, the fact that the fossil record calibration methods discussed above give estimates of the true time of origin of the first diagnosable fossilizable morphological feature of a lineage, not the divergence time from its sister group (**Figure 1A**). The reason is that the fossil record can only be used to constrain the time of origin of taxa where those taxa can be morphologically recognized as belonging to that lineage. Thus, paleontological methods only provide data on the morphologically diagnosable portion of a lineage’s history.

Often ΔT_Div-1stApo_ is probably relatively small, but it can be very large indeed. Below, I examine situations that span this range (see **Table 4** for a synopsis). I begin with the expectation with a complete fossil record.

**Table 4:** Relative magnitudes of ΔT_Div-1stApo_ (the difference between the paleontological estimate of the true time of origin of the first fossilizable apomorphy and the paleontologically unobservable lineage divergence time) and ΔT_Gap_ [the size of the gap between the first appearance in the fossil record (*FAD*) and the true time of origin of that first fossilizable apomorphy] (see Figure 1A).

Nature of the fossil record	ΔT_Div-1stApo_	Importance of ΔT_Div-1stApo_ compared with ΔT_Gap_
**Preservation potential ∼constant**		
Perfect—all species preserved	≤ a species duration	ΔT_Div-1stApo_ ≈ ΔT_Gap_ (section 4.1)
Good—many species preserved	≤ a species duration	ΔT_Div-1stApo_ < ΔT_Gap_ (section 4.2)
Poor—very few species preserved	≤ a species duration	ΔT_Div-1stApo_ << ΔT_Gap_ (section 4.3)
**Long, poorly preserved stem groups**	Can be many species durations	ΔT_Gap_ (section 4.4)
**Preservation decreases near base of clade**	Can be many species durations	ΔT_Div-1stApo_ can swamp ΔT_Gap_ (section 4.5)

### 4.1 Size of ΔT_Div-1stApo_ if All Extinct Species Were Preserved

Generally speaking, the appearance of a new fossilizable autapomorphy results in the recognition of a new paleontological species. Thus, if all extinct species have been preserved, and if there was no drop in fossilization potential prior to the emergence of the first apomorphy, the lag between the actual divergence time and the first appearance of the first diagnosable autapomorphy (ΔT_Div-1stApo_) will be in the order of an average paleontological species’ duration, because that is (very roughly) about how long it takes to evolve the first fossilizable diagnosable morphology:

(9) $$∆{\text{T}}_{\text{Div}-\text{1stApo}}\leq \text{average paleontological species duration}$$

However, if at the inception of the lineage there was rapid morphological change, then ΔT_Div-1stApo_ would be much shorter than a species duration; so, equation (9) should be viewed as an upper limit.

There are relatively few extant groups for which average species durations have been calculated, but for Cenozoic North American mammals, the average species duration is ∼2.3 million years (based on an analysis of >3,000 fossil species), while for planktic foraminifera, it is 5–10 million years, depending on their morphology; for angiosperms it is ∼3 million years, Coniferales at just over 5 million years, pteridophytes at ∼12 million years, and cycads at ∼15 million years [see compilation in Marshall, 2017].

Turning to the size of ΔT_Gap_, even if all species were preserved, there would still be a gap between the *FAD* of the founding species and the true time of origin of the first fossilizable apomorphy, but in all probability ΔT_Gap_ would be relatively small, less than an average species duration. Thus, to a first approximation, ΔT_Div- 1stApo_ and ΔT_Gap_ would probably be of a similar magnitude if all extinct species were preserved.

#### 4.1.1 Near-Perfect Fossil Records do Exist

A simple metric for measuring the quality of the fossil record is given by:

(10) Q=proportion of extant taxa found in the fossil record

Probably the richest fossil record is the marine skeletonized single-celled eukaryotic microplantkon. In particular, the Cenozoic macroperforate planktonic foraminifera are so abundant in the fossil record that not only does *Q* ≈ 1, but it is estimated that each species has at least an 81% of being sampled per million year interval (Ezard et al., 2011). In some geographic regions, marine macroinvertebrates are also well represented in the fossil record. For example, 77% of the 698 living species of bivalves and gastropods living at shelf depths in the California Province are found in the Pleistocene of the same region, with perhaps 85% (*Q* = 0.85) of all durable species captured in the fossil record (Valentine, 1989).

There is no comprehensive database of *Q* values, but within mammals, which overall have a strong fossil record, cetaceans are considered to have a good fossil record, with *Q* = 0.54 to 0.59 at the genus level (Quental and Marshall, 2010), while the primate fossil record is relatively weak, with *Q* < 0.07 at the species level (Tavaré et al., 2002). Didier et al. (2017) using an explicit diversification model estimate that about 14% (*Q* = 0.14) of all lineages of Permo-Carboniferous stem group mammal (synapsids) are currently known from the fossil record.

From a calibration standpoint, even if only 1% of extant taxa are preserved (*Q* = 0.01), there will still be many calibration points across a reasonably large phylogeny, given that species-turnover rates are sufficiently high that, on geologic timescales, the number of fossil species greatly exceeds the number of extant species. For example, for Cenozoic mammals, for each of the ∼5,500 living species, it has been estimated that there were ∼ 26 times that number that are now extinct (Marshal, 2017). Thus, for *Q* = 0.01, the expected number of preserved mammal species would be ∼5,500 x 0.01 x 26, or some 1,400 fossil taxa. If only one of the ∼5,500 extant mammal species was known in the fossil record (*Q* = 1/5,500 = 0.0002), we would still expect some 26 calibration fossils. Even if *Q* = 0.0, there may be fossils available for calibration (see orchid example, where *Q* = 0, Section 5.1 below).

### 4.2 Size of ΔT_Div-1stApo_ for Realistic (but Good) Fossil Records

As discussed in section 3.5 above, many species durations can be missing from the base of a taxon’s observed fossil record. Thus, for groups that are considered to have pretty good fossil records, the expected gap between the *FAD* and the time of appearance of the first fossilizable apomorphy (ΔT_Gap_) is likely to be many species durations, swamping the size of ΔT_Div-1stApo_, which is probably less than a species duration (equation [9]). For example, as discussed above, the oldest fossil crown group primate is about 55 million years old, but it is possible that the actual time of origin is ∼85 million years ago (for primate species, *Q* < 0.07). This difference of ∼30 million years is large compared with the ∼2.3 million years of an average mammalian species duration. Even if primates diverged from their nearest relatives at the upper limit of Wilkinson et al. (2011) range, at ∼69 million years ago, that is still ΔT_Gap_ of 14 million years, six average species-durations, much larger than <2 million years guesstimate for ΔT_Div-1stApo_.

Generally speaking, ΔT_Div-1stApo_ is likely to be small compared to ΔT_Gap_ for groups with good fossil records (except for most recently diverged clades, those that diverged on the order of a species duration ago): using the first fossilizable apomorphy as a proxy for the desired divergence time, i.e., ignoring ΔT_Div-1stApo_, will not typically add substantial error.

### 4.3 Size of ΔT_Div-1stApo_ for Weaker Fossil Records

For groups with poor fossil record, where very few species are preserved, the size of ΔT_Gap_ is even larger than with better fossil records, further decreasing the importance of ΔT_Div-1stApo_ over groups with better fossil records. Nonetheless, sometimes ΔT_Div-1stApo_ can be very large, as discussed below.

### 4.4 Size of ΔT_Div-1stApo_ With Long-Lived Poorly Preserved Stem Groups

#### 4.4.1 Neontological Data Have Significant Blind Spots

The pervasiveness of extinction has left large lacunae in the record of cladogenic events that can be accessed *via* the living biota. Those lacunae, unbroken branches on molecular phylogenies, can be very long and typically represent stem groups (diagrammed in **Figure 1A**). For example, the last common ancestor of all living birds, the base of the crown group, dates to the late Cretaceous, perhaps 66–87 million years ago (Benton et al., 2015), while the divergence between birds and their living sister group (the crocodiles) dates to deep in the Triassic or into the late Permian (247–260 million years ago) (Benton et al., 2015). Thus, there are no living lineages that connect to the bird lineage over the first ∼70% of its history, since it diverged from its nearest living relatives, the Crocodilia. Similarly, for angiosperms, where the fossil record is more difficult to work with (Coiro et al., 2019), the uncontested *FAD* for crown group angiosperms is ∼126 million years old (Coiro et al., 2019) with a maximum estimate of 256 million years ago (Barba-montoya et al., 2018), while their divergence from their living sister group is anywhere from 306 to 367 million years ago (Clarke et al., 2011)—perhaps 40% of the history of the angiosperm lineage is not accessible *via* living species.

#### 4.4.2 For Groups With Long Poorly Preserved Stem Groups ΔT_Div-1stApo_ Can Be Very Long

With relatively poor preservation potential, and with long stem groups, it can be very difficult in the fossil record to determine the size of the gap between the time it diverged from its living sister group and when the first diagnosable apomorphy of the group originated (ΔT_Div-1stApo_). For example, the primary paleontological diagnostic feature of angiosperms, tricolpate pollen, is first seen in the fossil record 125 ± 1 million years ago (Clarke et al., 2011; Coiro et al., 2019), while the *FAD* of its living sister group is known from rocks 307 ± 1 million years ago (Clarke et al., 2011). We don’t when the first species that we would recognize as an angiosperm by neontological criteria first appeared with in this interval, but ΔT_Div-1stApo_ could be well over a 100 million years.

#### 4.4.3 Mammalian Radiation and the End-Cretaceous Discontinuity

While fossil mammals were abundant before the end-Cretaceous mass extinction (Luo, 2007), it appears that there was an increase in the importance of mammals in terrestrial ecosystems after the mass extinction, accompanied by the relatively rapid evolution of new morphologies (Alroy, 1999). Thus, the ability to recognize members of the living mammalian orders may have been reduced in the Cretaceous if they were present—the ecological discontinuity across the end-Cretaceous mass extinction adds to the size of ΔT_Div-1stApo_ for the living mammalian orders, but we do not know how to quantify its magnitude.

### 4.5 Size of ΔT_Div-1stApo_ With a Radical Change in Preservability at the Base of the Clade

The size of ΔT_Div-1stApo_ can be very large if one or more of the earliest diagnostic features of the group dramatically increased the preservability of the lineage. For example, it appears that the last common ancestor of the well-skeletonized animal phyla was un-skeletonized—the first representatives of the animal phyla were probably not readily diagnosable in the fossil record (e.g., see Marshall, 2006; Marshall and Valentine, 2010). Thus, it is difficult to use the fossil record to assay how much before skeletonization the actual divergences between the phyla really were. Nonetheless, exceptional soft bodied preservation in rocks older than the first skeletonized phyla offers some maximum age constraints, although the difference between the minima and maxima is in the order of ∼85 million years (Benton et al., 2015).

Another group whose preservation potential appears to have changed dramatically during its history are the Scleractinian corals. Based on molecular clock data, it appears that their crown group extends in the Carboniferous, perhaps some 300 million years ago, well before the oldest fossils in the Middle Triassic, some 240 million years ago—the inference is that there was a substantial history where they were unskeletonized and therefore invisible in the fossil record (Romano and Palumbi, 1996), with more than one independent skeletonization event much later in the Triassic (Stanley, 2003).

## 5 Estimating Divergence Times for Groups With No, or Virtually No, Fossil Record—The Value of Hypothesis Testing

The entire discussion above on using the fossil record to constrain the absolute divergence times between lineages is predicated upon the assumption that the focal clade and, for some of the methods, the outgroups are known from at least several well diagnosed and dated fossils. However, for many groups, there is virtually no fossil record, or no fossil record at all. In these cases, well constrained calibrated timetrees are obviously difficult to obtain. Nonetheless, I want to make the case that hypothesis testing is still possible, especially if the minimum age estimate for a divergence time leads to a timetree that yields older dates than that proposed by the hypothesis—sometimes testing hypotheses is much less demanding of the data than trying to reconstruct the actual history of a group.

### 5.1 Virtually No Fossil Record

Paleobiologists typically work with groups with tens to tens of thousands of fossil species (e.g., trilobites are known from some 20,000 species). However, some groups are known from just a few species. Thus—for example, none of the 20,000–30,000 living species of orchid are known from the fossil record, and only three unequivocal extinct species are known from the fossil record (Ramírez et al., 2007; Conran et al., 2009). With such an awful fossil record, it is difficult to estimate reasonable maxima for the divergences within the orchids, or for the group as a whole (but see Section 3.4). Yet, even with the first fossil described, which has poor age constraints [anywhere from 15 to 20 million years, the degree of uncertainty associated with the difficulty of dating amber from the Dominican Republic (Iturralde-Vinent and MacPhee, 1996)], it was possible to establish that the orchid crown group extends into the Cretaceous, refuting the hypothesis of Cenozoic origins (Ramírez et al., 2007). This result was obtained by simply using the fossil to date one node in an ultrametric tree, a result further supported in a Bayesian analysis using all three fossils (Gustafsson et al., 2010).

### 5.2 With No Fossil Record

Particularly at lower taxonomic ranks, many groups have no fossil record, neither do their immediate outgroups. Nonetheless, despite the lack of direct temporal data, average rates of molecular evolution estimated for closely related groups can sometimes provide valuable temporal data. For example, using an insect-wide molecular rate of ∼1.5% change/million years for the mitochondrial COI gene, Quek et al. (2007) were able to refute the hypothesis that diversification of mitochondrial lineages of *Crematogaster* ants from the Sunda Shelf (peninsular Malaysia, Borneo, Sumatra, Java, etc.) was driven by the glacial-interglacial cycles that repeatedly exposed and drowned the Sunda Shelf over the last million years. Instead, it appears that the ant lineages diverged from 1 to 20 million years—the COI gene would have had to have evolved ∼10 times faster than the 1.5% rate to support the glacial-interglacial hypothesis.

#### 5.2.1 Almost All Clades Are Embedded in More Inclusive Clades That Have a Fossil Record

Almost all clades, at least within animals and plants, lie within more inclusive clades where minimum and maximum age constraints are available (e.g., see Benton et al., 2015). Thus, at some level, temporal constraints can always be found for most groups, even if the dating precision might be low within the unfossiliferous ingroup.

## 6 Summary

The quality of temporal calibration is highly variable, depending on the group and the fossil record available. Nonetheless, some generalizations can be made as a function of the age of the group, and its correlates, the group’s geographic range and species richness (**Table 5**). If care is taken with the paleontological calibrations themselves, and with judicious analysis of data with multiple approaches, robust timetrees are well within our grasp for many taxa.

**Table 5:** Relative magnitude of major factors that challenge our ability to estimate robust soft maxima on divergence times.

	Temporal, geographic, and taxonomic scale		
	Shallow time	Deeper time	Deepest time
	< ∼2 million yrs	∼2 to ∼540 million yrs	> ∼540 million yrs
	Quaternary	Pliocene–Cambrian	Precambrian
	∼Local scale	Regional to global scale	∼Global scale
	∼Genus	∼Family, order, class	∼Phylum, kingdom
**Coalescence**	Often large	Typically unimportant, less than 1% the age of the clade	Unimportant
**Dating errors (radiometric dates; biostratigraphy)**	Can be large, but typically small	Typically unimportant, less than 1% the age of the clade	Can be important due to lack of effective biostratigraphy
**ΔT** **_Gap_**	Can be very large; the fossil and rock record is often very spotty at this timescale	Small to large (depends on the group)	Typically large
**ΔT** **_Div-1stApo_**			
Preservation ∼constant	Similar to ΔT_Gap_	Smaller or much smaller than ΔT_Gap_	N/A—most groups have changed their preservation potential
Preservation drops at clade base	N/A—most groups this young didn’t change their preservation potential	Can be much larger than ΔT_Gap_	Typically much larger than ΔT_Gap_

## Author Contributions

CM is sole author, and thus was responsible for all of its components.

## Funding

This work was partially supported by the Philip Sandford Boone Chair in Paleontology at the University of California, Berkeley.

## Conflict of Interest

The author declares that the research was conducted in the absence of any commercial or financial relationships that could be construed as a potential conflict of interest.

## Appendix A Relationship Between the Number of Fossil Localities and Age of the *FAD* of a Taxon, and the Mean and Variance of the Equivalent Lognormal Prior

Following Section 3.1.2.1, the desired lognormal prior should have a mode, $e^{\left(\mu -\sigma^{2}\right)}$, which extends an average gap size (*FAD*/*n*) below the oldest fossil (*FAD*). It should also have a 95% tail, equivalent to the *p* = 0.95 quantile, $\;e^{\left(\mu +\sqrt{2}\sigma erf^{-1}\left(2p-1\right)\right)}$, extending* FAD*(1 – *p*)^–1/^ *^n^* beyond the age of the *FAD*. Thus, we have two unknowns (µ and σ^2^) and two equations, one for the mode and one for the 95% quantile:

(A1) $$e^{\left(\mu -\sigma^{2}\right)}=FAD/n$$

(A2) $$e^{\left(\mu +\sqrt{2}\sigma erf^{-1}\left(2p-1\right)\right)}=FAD{\left(1\;–p\right)}^{-1/n}$$

Taking natural logarithms, (A1) and (A2) become, respectively:

(A3) $$\mu -\sigma^{2}=\ln\left(FAD\right)-\text{ln}\left(n\right)$$

(A4) $$\mu +\sqrt{2}\sigma erf^{-1}\left(2p-1\right)=\ln\left(FAD\right)-(\ln\left[1-p\right])/n$$

Rearranging equation (A3) gives the mean, µ:

(A5) $$\mu =\ln\left(FAD\right)-\ln\left(n\right)+\sigma^{2}$$

which is equation (6) in the text. We now need an expression for the variance, σ^2^. This can be found by substituting equation (A5) into equation (A4). With some re-arranging we have:

(A6) $$\sigma^{2}+\sigma \sqrt{2}erf^{-1}\left(2p-1\right)+(\text{ln }\left[1-p\right]/n-\text{ ln}\left[n\right])=0$$

We can solve for σ using the quadratic formula:

$$\sigma =\left(-b\;\pm \sqrt{b^{2}-4ac}\right)/2a\;$$

where *a* = 1, $b=\sqrt{2}erf^{-1}\left(2p-1\right)$and *c* = (ln)[1 – *p*]/ *n* – ln[*n*]). Given that *erf* ^–1^(2*p* – 1) = 1.163087 and ln (1– *p*) = –2.9957 for *p* = 0.95, we have:

$$\begin{array}{l} \sigma =-\sqrt{2}\;\left(1.163087\right)/2\;\pm \\ 0.5\sqrt{{\left(\sqrt{2}\;\left(1.163087\right)\right)}^{2}-4(-2.9957\;/n-\text{ ln}\left[n\right])} \end{array}$$

After simplification and squaring, we arrived at equation (7) in the text:

(A8) $$\sigma^{2}={\left(-0.8224+0.5\sqrt{2.7055-4(-2.9957\;/n-\text{ ln}\left[n\right])}\right)}^{2}\;$$

The decision to add rather than subtract $\sqrt{b^{2}-4ac}$ was based on numerical tests to determine which of the two options returned the correct mode and 95^th^ percentile.
